# Supramolecular Complexation of Carbohydrates for the Bioavailability Enhancement of Poorly Soluble Drugs

**DOI:** 10.3390/molecules201019620

**Published:** 2015-10-27

**Authors:** Eunae Cho, Seunho Jung

**Affiliations:** 1 Center for Biotechnology Research in UBITA (CBRU), Institute for Ubiquitous Information Technology and Applications (UBITA), Konkuk University, 120 Neungdong-ro, Gwangjin-gu, Seoul 05029, Korea; E-Mail: echo@konkuk.ac.kr; 2 Microbial Carbohydrate Resource Bank (MBRC), Konkuk University, 120 Neungdong-ro, Gwangjin-gu, Seoul 05029, Korea; 3 Department of Bioscience and Biotechnology, Konkuk University, 120 Neungdong-ro, Gwangjin-gu, Seoul 05029, Korea

**Keywords:** poorly soluble drugs, carbohydrates, supramolecular complexation, bioavailability enhancement

## Abstract

In this review, a comprehensive overview of advances in the supramolecular complexes of carbohydrates and poorly soluble drugs is presented. Through the complexation process, poorly soluble drugs could be efficiently delivered to their desired destinations. Carbohydrates, the most abundant biomolecules, have diverse physicochemical properties owing to their inherent three-dimensional structures, hydrogen bonding, and molecular recognition abilities. In this regard, oligosaccharides and their derivatives have been utilized for the bioavailability enhancement of hydrophobic drugs via increasing the solubility or stability. By extension, polysaccharides and their derivatives can form self-assembled architectures with poorly soluble drugs and have shown increased bioavailability in terms of the sustained or controlled drug release. These supramolecular systems using carbohydrate will be developed consistently in the field of pharmaceutical and medical application.

## 1. Introduction

Poorly soluble drugs belong to Biopharmaceutics Classification System (BCS) class II (low solubility/high permeability) and IV (low solubility/low permeability) materials [1]. The poor aqueous solubility of drugs has been a main obstacle in drug discovery and development, since it results in poor bioavailability at the active site [2]. With increasing molecular weight and log *p* values, the solubility of drugs decreases. Actually, 40% of drug candidates have been listed as practically insoluble (<100 μg/mL), whereas only 8% of new drug candidates have shown both high solubility and permeability [1,3]. To enhance the bioavailability of insoluble drugs, various techniques, including use of co-solvents, micronization, salt formation, and supramolecular complexation have been reported [4,5]. In the case of most non-intravenous administrations, the bioavailability is much less than 100%, because all drugs may not be adsorbed and metabolized before the delivery at the target site. This review aims to shed light on the supramolecular complexation of drugs with carbohydrates for bioavailability enhancement with flexibility and simplicity in the design and resulting in a considerable increase in the solubility and dissolution of drugs.

Supramolecular chemistry is defined as “chemistry beyond the molecule” and its importance has been established by the award of the 1987 Nobel Prize in chemistry [6]. For biochemical interactions, the classical lock-and-key model has been changed and extended. Biomolecular architectures such as proteins, DNA, and bio-membranes are rather formed by supramolecular interactions, and they regulate the biological processes [7,8]. A lot of bioinspired materials have also been developed using supramolecular chemistry [9]. Self-assembly, molecular recognition, metal coordination, folding, and host-guest chemistry are the sophisticated concepts of supramolecular chemistry [10,11]. They are mainly composed of various noncovalent interactions including hydrogen bonding, dipole-dipole interactions, van der Waals forces, pi-pi interactions, and electrostatic interactions between molecules [12].

Hydrogen bonding plays crucial roles in biological molecular recognition together with other noncovalent interactions [13]. Carbohydrates (C_n_H_2n_O_n_), the key molecules in Nature, are polyhydroxyaldoses or ketoses, which are the possible hydrogen bonding donors and/or acceptors. They are also coated onto all the cells and involved in various types of supramolecular interactions. Through cooperative hydrogen bonding, carbohydrate recognition mediates cell-cell interactions, pathogenesis, and immune responses [14,15]. To the supramolecular chemist, the recognition ability of carbohydrates can be useful for the biomedical application such as drug delivery and development. Therefore, carbohydrate-based drug development has grown rapidly as a promising and exciting research field. In particular, the supramolecular association of carbohydrates with drugs could enhance the bioavailability of poorly soluble drugs. The strategies are varied, depending on the carbohydrate types such as monosaccharides, oligosaccharides, and polysaccharides.

## 2. Monosaccharides

### 2.1. Prodrug System

Using monosaccharides, drug-monosaccharide conjugates can be used as a type of prodrug. An anticancer drug, dodetaxel, was conjugated with the glucose moiety, resulting in 52-fold increase in its solubility compared to the original drug [16]. Glucuronide prodrugs of doxorubicin and glycosyllonidamine have been reported [17,18,19]. Several antitumor agents such as bleomycin, anthracycline, and mithramycin also have a glycosidic moiety in their intrinsic structures. Their water solubility can be increased by the modulation of their hydrophilicity, and the glycosylated drugs can take advantage of the recognition by glucose transporters [20]. As another example, when a neurotransmitter for antiparkinsonian agents is glycosylated, the glycoconjugate could be delivered into the central nervous system across the blood brain barrier [21,22]. Some peptide drugs have been glycosylated, and their physicochemical and pharmacological properties also changed [23,24]. They undergo enzymatic hydrolysis in plasma, and the free drugs can be released and become active for *in vivo* investigation.

### 2.2. Glycosylated Carrier

For drug delivery, various carriers such as liposomes, micelles, dendrimers, micro/nanoparticles, and micro/nanocapsules are available [25,26,27]. The use of monosaccharides offers the characteristic effects for the advanced carrier system of poorly soluble drugs [28]. Since carbohydrate binding proteins are present on different cell surfaces, glycosylated carriers can be designed for targeted delivery [29]. Furthermore, galactose-, fucose-, and mannose-conjugated carriers show bioadhesive properties, stability/solubility enhancement, and reduced immunogenicity/toxicity [30,31,32]. In terms of solubility of poorly soluble drugs, the limited effect of monosaccharides can be overcome by the use of oligosaccharides.

## 3. Oligosaccharides

### 3.1. Binary Systems

#### 3.1.1. Cyclic Oligosaccharides and the Derivatives

**Figure 1:**
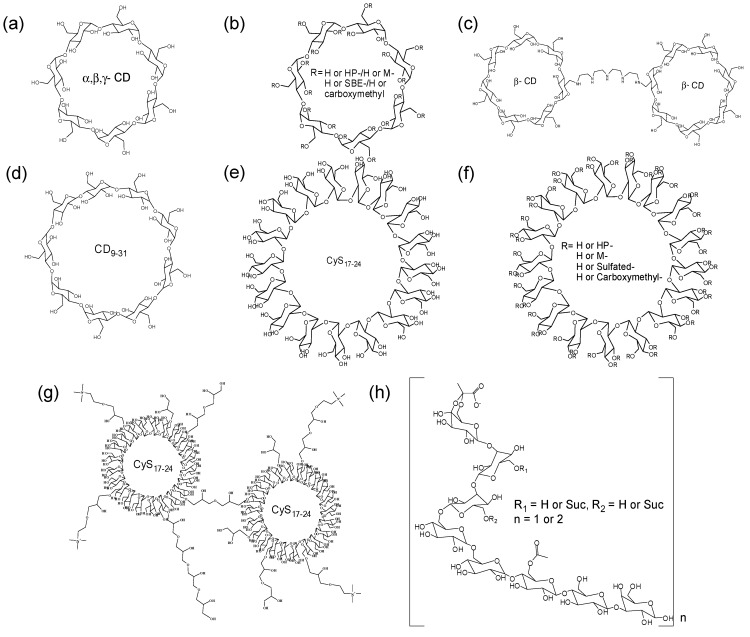
Oligosaccharide hosts in binary system. (**a**) CD; (**b**) CD derivatives; (**c**) CD dimer; (**d**) Large CD; (**e**) CyS; (**f**) CyS derivatives; (**g**) Cys dimer; and (**h**) Linear oligosaccharides.

Cyclodextrin (CD): In 1896, the use of CD glucosyltransferase (CGTase) allowed a practical production of CD from starch. CDs are cyclic α-d-1,4 glucans, most commonly composed of 6, 7 and 8 glucosidic units called α-, β-, and γ-CD, respectively (Figure 1a). The macrocycles can maintain a conformationally constrained structure compared to linear structures. The respective cavity diameters of α-, β-, and γ-CDs are in the ranges 4.7–5.3, 6.0–6.5, and 7.5–8.3 Å, and the height of the torus is 7.9 Å in all types [33,34]. In 1911, the first noncovalent host-guest inclusion complexation of CD and other molecules was reported, and the first drug/CD patent was disclosed in 1953 [35,36]. With the characteristic cone or torus shape, various drugs containing ibuprofen, itraconazole, diclofenac, or atenolol have been incorporated into the cavity of β-CD even now, with a significant enhancement in the stability or solubility [37,38,39,40]. This is reasonable because CD can prevent crystallization, increase the dissolution of hydrophobic drugs, and protect the degradation of labile drugs by shielding guests with the hydrophobic cavity and the external layer of hydrophilic carbohydrates. They may also decrease the drug toxicity by making the drug more effective at lower doses. Furthermore, the native CD has Generally Regarded as Safe (GRAS) status in the United States and is found in many pharmaceutical and biomedical products.

CD derivatives: β-CD is generally used owing to its easy availability and appropriate size, however its solubility in water is relatively low (1.8%) because of the intramolecular hydrogen bonding and strong order on the surrounding water [41]. To improve the complexation ability and aqueous solubility, CD derivatives have been synthesized (Figure 1b), and the aqueous solubilities of hydroxypropyl (HP-), methyl (M-), and sulfobutylether (SBE-) β-CDs are >60%, 50%, and 50%, respectively [34]. Using HP-β-CD, nonpolar drugs such as itraconazole, indomethacin, hydrocortisone, cisapride, and mitomycin were marketed in the complex form in Europe and the United States [42,43]. HP-β-CD is also much more toxicologically benign than the natural β-CD [44]. In the case of M-β-CD or SBE-β-CD derivatives, the antibacterial chloramphenicol or the antifungal voriconazole have been introduced as complex solution formulations. However, because M-β-CD has hemolytic activity based on the complexation with cholesterol from membranes, its oral administration is limited [45]. Using ionic interactions, carboxymethyl- and (2-hydroxy-3-(trimethylammonio)propyl) β-CDs have been investigated for their solubilizing effect with oppositely charged drugs [46]. Finally, dimeric or oligomeric β-CD have attracted much attention as versatile receptors for molecular recognition [47]. Two or more hydrophobic complexation sites can enhance the stability, selectivity, and flexibility towards various nonpolar drugs. For example, the complexes of titanocene or paclitaxel with CD dimer are fairly soluble in water (Figure 1c), and the bioavailability is dramatically improved compared to the original drug [48,49].

Large CDs: The large CD comprising more than eight glucose units was isolated from the reaction mixture of CGTase and starch in 1957 by French and co-workers (Figure 1d) [50]. The CDs containing nine to 13 glucoses are sequentially called as δ-, ε-, ζ-, η-, and θ-CD. δ-CD (CD_9_) is found to be not a doughnut-shaped but rather elliptic boat-shaped, and has shown a solubilization effect for large guests such as digitoxin and spironolactone [51,52]. CD_10_, CD_14_ and CD_26_ also display distorted structures containing double band-flip motifs owing to their inherent flexibility [53,54]. Although the complex itself is less stable than the rigid α-, β-, and γ-CDs, the flexible complex may be better for the effective release of drugs [55]. Furthermore, a variety of cavity sizes can be useful for special guests not geometrically suitable for α-, β-, and γ-CDs.

Cyclosophoraoses (CyS) and the derivatives: CyS isolated from *Rhizobium* species are cyclic β-1,2 glucans containing 17 and 40 glucose residues (Figure 1e). In 1984, CyS_17_ were found to exhibit the complexation ability with hydrophobic guests including indomethacin, propericiazine, reserpine, and steroids [56]. The cavity was considered to be able to accommodate three-dimensionally extended guest molecules, and the diameter and depth of the cavities were estimated as 9 and 15 Å according to the CPK models. Besides, CyS_17-24_ efficiently complexed with paclitaxel, indomethacin, or luteolin [57,58,59]. The molecules are shaped such as a distorted and flexible ring, and the possibility of a different molecular mechanism for the complexation from the typical inclusion complexation by CD was suggested [60,61]. They could also be substituted by various functional groups including carboxymethyl, hydroxypropyl, sulfated, or methyl groups (Figure 1f) [62,63,64,65]. CyS oligomers were also designed (Figure 1g), and the cooperative complexation enhanced solubility and bioavailability of fisetin [66]. Because the functionalized CyS_17-24_ provide the additional space and property for guests, they are expected to behave as promising solubilizers for poorly soluble drugs.

#### 3.1.2. Linear Oligosaccharides

Further, host-guest complexation was not found to be the exclusive property of cyclic structures. In 1989, it was demonstrated that cell-cell recognition is initiated by direct oligosaccharide-oligosaccharide interactions, indicating hydrophobic effects are responsible for the interaction [67]. In carbohydrate-protein interactions, stacking and non-polar interactions between sugar and tryptophan or phenylalanine residues of proteins were also observed, together with hydrogen bonds or metal coordination [68]. From this point of view, the hydrophobic character of linear carbohydrates was investigated with fluorescence probes, and the host-guest complexation might involve an induced-fit type adjustment [69]. Non-cyclic hexadecasaccharide has been reported as an effective complexing agent for pyrimethamine, haloperidol, or isoflavonoids (Figure 1h) [70,71,72]. In fact, the solubility of the antimalarial drug pyrimethamine and antipsychotic medication haloperidol was increased 42- and 87-fold in water by complexing with succinoglycan dimer D3 [70,71]. Even in octasaccharides, a solubility enhancement of pindolol was described, and their cytotoxic effect was low enough to warrant further study [73]. Recently, an acyclic cucurbit[*n*]uril molecular container has also been reported to overcome potential limitations such as the structural rigidity and slow dissociation kinetics of macrocyclic cucurbit[*n*]urils [74]. Acyclic oligosaccharides would be flexible hosts to recognize a broad range of poorly soluble drugs and provide high dissociation kinetics.

#### 3.1.3. Preparation of Inclusion Complexes

To overcome the practical problems, including long processing time, the use of excessive solvent, and multistep synthesis, practical preparation methods have been developed as follows. The preparation can be optimized depending on the substrates.

Kneading [75]: In this method, carbohydrate hosts were placed in a mortar and wetted with little amount of water or hydro-alcoholic solution. Subsequently, the drug was added to the host paste, and the mixture was kneaded for a specified time. Finally, the kneaded sample was dried.

Freeze-drying [76,77]: Drugs and carbohydrate hosts were dissolved in water to achieve equilibrium, and the solutions were frozen by immersion in liquid nitrogen, and the frozen solutions were lyophilized.

Spray-drying [78]: Aqueous solutions of carbohydrate hosts and alcoholic solution of drugs are mixed to produce a clear solution. The solution is then spray-dried using a spray dryer.

#### 3.1.4. Analysis of Inclusion Complex

1. UV-Vis spectroscopy—Measurement of drug solubility enhancement by complexation.
2. Nuclear Magnetic Resonance (NMR) spectroscopy—^1^H-NMR is a suitable method for the evaluation of noncovalent interactions at the molecular level [79]. To elucidate the intermolecular interaction of inclusion complexes, two-dimensional NMR spectroscopy (Nuclear Overhauser enhancement spectroscopy (NOESY) or Rotating frame nuclear Overhauser effect spectroscopy (ROESY)) has also been frequently used, because two protons located within 5 Å induce an NOE crosspeak [80].
3. Thermogravimetric Analysis (TGA)/Differential Scanning Calorimetry (DSC)—TGA curves describe the weight losses of pure components and the complexes. DSC is performed to characterize the solid-state interactions for the inclusion complexes as compared to the melting points [81].
4. Fourier Transform Infrared (FTIR) spectroscopy—The analysis of the vibrational changes upon the inclusion of drugs with a host.
5. X-ray Powder Diffractometry (XRPD)—The powder diffraction patterns of drugs and complex are compared. In principle, drugs displayed sharp peaks, which are the characteristics of an organic molecule with crystallinity, and the complex shows different patterns with crystalline drugs.
6. Scanning Electron Microscopy (SEM)—The surface morphology of the pure and complexed form is investigated. The morphological changes are frequently analyzed to evaluate the interaction between drugs and host [82,83].
7. Electrospray mass spectrometry (ESI-MS)—Determination of molecular association of noncovalent bonding.
8. Computational method (Molecular modeling)—The appropriate binding mode of complex between host and drug can be derived from molecular docking simulations [84].

#### 3.1.5. Phase Solubility Studies

**Figure 2:**
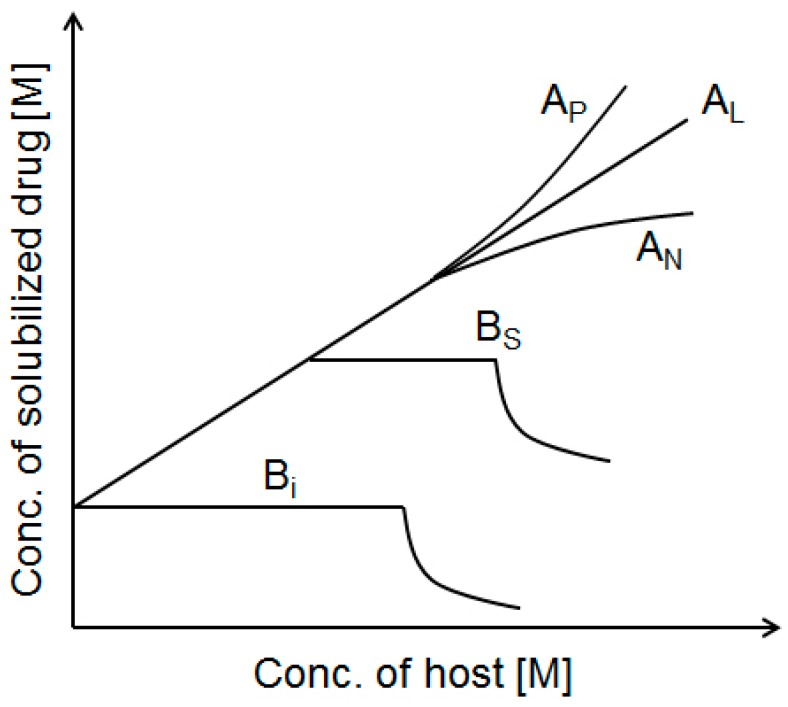
Phase solubility diagram.

The complexation between drugs and carbohydrates is caused by forming dynamic non-covalent bonds, which increases the aqueous solubility of drugs. In phase-solubility diagram (Figure 2), the solubility enhancement of drugs is assessed as a function of host concentration [85]. Based on the shape of the plot, the diagram is classified as A_P_, A_L_, A_N_, B_S_, or B_i_ [42].

A type profile: In this profile, the apparent solubility of drugs increases as a function of host concentration. The A_L_ graph indicates a linear increase in solubility, whereas A_P_ and A_N_ type graphs indicate a positive and negative deviation from linearity, respectively. Although A_N_ systems are not easy to interpret, A_P_ type suggests the formation of a higher-order complex with respect to the host. In A_L_ type, a plot of drug concentration (D_t_) *vs.* host concentration (H_t_) for the formation of D_m_-H complex gives a linear line with the intercept and the slope is defined as:

(1) $$\text{slope}=\text{mK}S_{0}^{m}/(1+\text{K}S_{0}^{m})$$

where, if m is known, the K value can be calculated from the slope and intercept (S_0_, the intrinsic solubility of the drug in water).

If the complex has a 1:1 drug:host ratio, the complexation is an equilibrium governed by an equilibrium constant (K_1:1_) calculated based on the linear phase-solubility profiles (A_L_ type) using the following equation:

K_1:1_ (M^−1^) = slope/S_0_(1 − slope) (2)

In this case, the slope is always less than unity [86]:

In the case of 2:1 drug:host complexation, the slope of the A_L_ type diagram is less than two, and the association constant (K_2:1_) is determined by the following equation [87]:

(3) $${\text{K}}_{2:1}({\text{M}}^{-1})=\text{slope}/S_{0}^{2}(2-\text{slope})$$

B type profile: When the system forms complexes with limited solubility, the diagram is B type, which is usually observed with β-CD. B_S_ type is derived from the initial soluble complex and insoluble complex at the maximum point. B_i_ style indicates that the complex is too insoluble to increase the isotherm.

#### 3.1.6. Drug Delivery and CD Elimination from the Drug/CD Complexes

Complexation certainly involves the issue of the mechanism(s) of drug release. The answer is thought to be dissociation owing to dilution, protein binding, tissue uptake of drugs, competitive displacement of drugs from the complex, and host elimination [88,89]. These drug/host complexes may alter drug metabolism route, biodistribution, and tubular reabsorption, and thereby the bioavailability of drugs can be changed. In particular, myricetin [90], isotretinoin [91], benznidazole [92], and etodolac exhibit 9.4-, 4.7-, 3.6-, and 2.5-fold increases in bioavailability upon addition of drug/CD derivative complexes for their oral administration [93]. As well as, the CDs are ultimately digested by bacteria in the gastrointestinal tract or colon to monosaccharides or gases including carbon dioxide, hydrogen, and methane [94].

### 3.2. Ternary Systems

Ternary systems have been designed to improve the shortcomings of the above binary systems. In general, the main property of carbohydrate hosts is the modification of the physicochemical and biological characteristics of poorly soluble drugs by complex formation. Using another factor, ternary complexes could be more efficient for the solubility and bioavailability of drugs with the use of a small amount of carbohydrates. As modifiers, organic acids such as ascorbic acids, citric acids, and tartaric acids are used together with β-CD, and they can modulate the pH under aqueous conditions [95,96]. Accordingly, the solubility, stability, and phase solubility types are simultaneously affected. Besides, arginine, co-solvent, and a suitable water-soluble polymer also provided a synergistic effect for improving drug solubility in the presence of CD [97,98,99]. By adding the third auxiliary substance, lecithin, the solubility, dissolution, and stability of drugs were better than that those of the original drugs and binary systems [100]. Based on the amphiphilic properties of lecithin, the hydrophobic interactions with drugs, hydrogen bonding with HP-β-CD, and the cooperative advantage is greater than the simple statistical contributions of individual components.

### 3.3. Multinary Systems

#### 3.3.1. CD Amphiphiles

CD can even be conjugated to lipid molecules, and the amphiphile forms a micellar or vesicular system via self-assembly [101,102,103,104]. The resulting structure provides a higher drug loading, stable colloidal system, and controlled drug release. The first amphiphilic CDs were synthesized by Kawabata *et al.*, in 1986, and the alkylsulfinyl β-CD showed interesting molecular assembly properties [105]. Recently, anthraquinoyl-, hexadecyl-, sulfated hexanoyl-modified β-CDs were developed for the delivery of anticancer (paclitaxel, docetaxel), and antiviral (acyclovir) drugs [106,107,108]. Supramolecular amphiphiles are of significant interest as hybrid materials with inclusion capacity and nanoparticulate properties [101].

#### 3.3.2. CD Pendent Polymers

At the same time, CD-containing polymers have been explored for novel drug delivery platforms and are more suitable in medication for parenteral adminstration than the natural β-CD [109,110]. The systems consist of the polymeric, assembled network, and host-guest complexation, allowing the effective loading and controlled release of drugs. Depending on the crosslinkers, soluble or insoluble β-CD polymers are obtained. Hyper-crosslinked CDs were first called nanosponges in 1998 [111], and insoluble polymers have been used as excipients in preparing tablets, suspensions, and capsules for drug carriers with soluble CD polymers [112,113]. For doxorubicin delivery to treat some leukemias and cancers, quaternary ammonium crosslinked β-CD and β-CD-centered amphiphilic polymers were achieved [114,115]. The three-dimensional architecture of CD pendent polymers usually forms hydrogels or nanoparticles, which are potentially superior in the biomedical and pharmaceutical fields.

## 4. Polysaccharides

Polysaccharides are polymeric carbohydrate molecules composed of monosaccharide units bound together by glycosidic linkages. They vary depending on their sources and structural units, and are easily available, inexpensive, eco-friendly, and biocompatible materials. Based on their primary sequence, they can adopt certain shapes (secondary structures like ribbons and helices) and the ordered specific structure (tertiary structures of multiple ribbons and helices) formed via energetically favorable interactions [116]. Sometimes, quaternary structures have shown higher levels of organization. Using these supramolecular polysaccharides, multi-dimensional carrying systems were prepared to control the drug release in a desirable manner.

### 4.1. Polysaccharide Drug Conjugates

In 1975, the concept of polymer-drug conjugates was introduced for the first time for the delivery of hydrophobic drugs to their sites of action [117]. The conjugates alter the biodistribution and circulation time of the original drugs. So far, hyaluronic acid [118,119], dextran [120], chitosan [121], heparin [122], alginate [123], pullulan [124], arabinogalactan [125,126], and starch have all been developed as drug conjugation platforms [127]. Hyaluronic acid is an anionic polysaccharide containing alternating disaccharide units of d-glucuronic acid and *N*-acetyl-d-glucosamine linked by β-1,4 glycosidic linkages. In the structure, the hydroxyl and carboxylic acid groups provide the appropriate sites for the conjugate. Because it has a strong affinity for the cancer cell marker, CD44, the selective adsorption is an additional advantage for the anticancer bioavailability [128]. Dextran is a glucan with mostly α-1,6 glycosidic linkages and has been approved as a plasma expander [129]. Chitosan is a biopolymer of β-(1-4)-linked d-glucosamine and *N*-acetyl-d-glucosamine, and the reactive amine group is important for chemical modification. The muco-adhesive property and the insolubility at pH 7.4 are considered for the development into advanced materials. Heparin is a highly sulfated glycosaminoglycan and has been used as the anticoagulant. Alginate is a linear copolymer of β-1,4-mannuronate and α-guluronate, and its gel-forming ability via calcium binding is a characteristic property. Pullulan is a neutral polysaccharide composed of maltotrioses linked by α-1,4 and α-1,6 bond types, and starch consists of helical amylose and branched amylopectin. Arabionogalactan is a heteropolymer consisting of arabinose and galactose. Among these, hyaluronic acid, carboxymethyl dextran, and oxidized dextran conjugates have entered clinical trials [118,130,131,132], showing improved adverse reaction profiles, enhanced therapeutic efficacy, and target-oriented properties.

### 4.2. Supramolecular Architectures for Polysaccharide Drug Carriers

**Figure 3:**
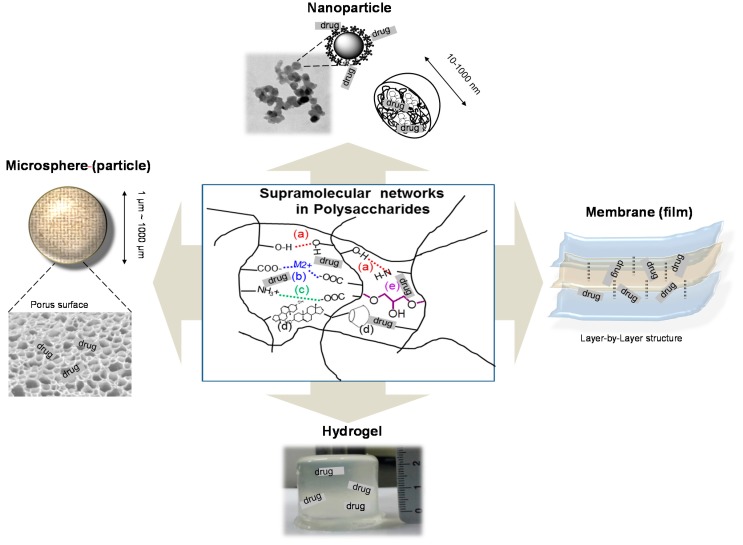
Polysaccharide-based advanced materials and supramolecular architectures for biomedical applications. The supramolecular architecture is formed by noncovalent methods such as hydrogen bonding (**a**); metal coordination (**b**); ionic interaction (**c**); hydrophobic interaction (**d**); and covalent methods using cross-linkers (**e**).

Polysaccharides provide supramolecular architectures derived from cooperative intra- and interchain association by hydrogen bonding, dipole and ionic interactions, and solvation (Figure 3). Supramolecular structures have distinct structural characteristics and functions that are not shown in monosaccharide units. Furthermore, their reactive groups (hydroxyl, amino, and carboxylic acid) can be easily modified, and various polysaccharide derivatives have been synthesized as novel supramolecular architectures. The driving forces for the three-dimensional structures are classified as noncovalent and covalent methods (Table 1). Supramolecules can provide the high encapsulation efficiency and cell internalization, decrease side effects, and protect the liable drugs via supramolecular complexation. As a result, they are designed for controlled drug release to maintain a constant drug concentration for the desired time with minimum side effects.

**Table 1:** Polysaccharide-based carriers for the bioavailability enhancement of poorly soluble drugs.

Supramolecular Forces		Used Polysaccharides	Architecture Types	Drugs	References
Non-covalent bond	Hydrogen bond	Hydrolyzed xyloglucan	Hydrogel	Ondansetron Indomethacin Mytomycin C	[133,134,135,136]
		Hydroxypropyl Methylcellulose	Hydrogel	Indomethacin	[137]
	Metal coordination	Alginate-calcium ion	Nanoparticle	Rifampicin, Doxorubicin	[138,139]
		*N*-succinyl chitosan-alginate	Hydrogel	Nifedipine	[140]
		Alginate-calcium carbonate	Hydrogel	Ibuprofen	[141]
	Ionic interaction	Chitosan-tripolyphosphate	Nanoparticle	Ciprofloxacin	[142]
		Chitosan-tripolyphosphate-hydroxypropylcyclodextrin	Nanoparticle	Furosemide, Triclosan	[143]
		Chitosan-tripolyphosphate-dextran sulfate	Microsphere	Ibuprofen	[144]
		Chitosan-dextran sulfate	Nanoparticle	Amphotericin B	[145]
		Chitosan-glycyrrhetic acid	Nanoparticle	Glycyrrhetic acid	[146]
		Chitosan-β-glycerophosphate	Hydrogel	Paclitaxel	[147]
		Carrageenan Dextran sulfate	Nanosphere	Ciprofloxacin	[148]
	Hydrophobic interaction	Ceramide modified hyaluronic acid	Nanoparticle	Docetaxel, Doxorubicin	[149,150]
		Deoxycholic acid modified hyaluronic acid	Nanoparticle	Paclitaxel	[151]
		Histidine modified hyaluronic acid	Nanoparticle	Doxorubicin	[152]
		Pullulan acetate	Nanoparticle	Silymarin	[153]
		Cholesterol modified chitosan	Nanoparticle	Epirubicin	[154]
		Deoxycholic acid-modified chitosan	Nanoparticle	Adriamycin, Doxorubicin	[155,156]
		5β-cholanic acid modified chitosan	Nanoparticle	Paclitaxel, Camptothecin	[157,158]
		Stearic acid-*g*-chitosan	Nanosphere	Doxorubicin	[159,160]
		*N*-acetyl histidine-conjugated glycol chitosan	Nanoparticle	Paclitaxel	[161]
		Cholic acid modified dextran	Nanosphere	Indomethacin	[162]
		CD polymer-dextran polymer	Nanogel	Benzophenone, Tamoxifen	[163]
		Acetylated chondroitin sulfate	Nanogel	Doxorubicin	[164]
Covalent bond	Cross-linker or Copolymer	Chitosan (glutaraldehyde, sulphuric acid)	Microsphere	Diclofenac, Docetaxol Clozapine	[165,166,167]
		Polyacrylamide-*g*-chitosan copolymer	Microsphere	Nifedipine	[168]
		Chitosan-Pluronic copolymer	Nanoparticle	Indometacin, Doxorubicin	[169,170]
		Pullulan-*g*-poly(l-lactide) copolymers	Hydrogel	Doxorubicin	[171]
		Poly(dl-lactide-co-glycolide)-grafted pullulan	Nanosphere	Adriamycin	[172]
		Cellulose-*graft*-poly(l-lactide) copolymers	Nanosphere	Paclitaxel	[173]
		Dextran-*b*-poly(DL-lactide-coglycolide) copolymer	Nanosphere	Doxorubicin, Amphotericin B	[174,175]
		Poly[lactic-co-(glycolic acid)]-grafted hyaluronic acid copolymer	Nanoparticle	Doxorubicin	[176]
		Dextran-*b*-poly(ε-caprolactone)	Nanoparticle	Doxorubicin	[177]
		Chondroitin sulfate-Pluronic copolymer	Nanoparticle	Doxorubicin	[178]
		Starch (epichlorohydrin)	Microsphere	Ampicillin	[179]
		Hyaluronic acid (1,3-diaminopropane)	Hydrogel	Ibuprofen	[180]

Among non-covalent bonds, hydrogen bonding is basically involved to make the supramolecular association of polysaccharides. With the same β-1,4-d-glucan backbone, xyloglucan and cellulose have ribbon-like shapes, and the modified structures can form hydrogel structures based on non-covalent interactions [133,134,135,136,137]. Carboxyl (–COOH) groups in alginate are able to interact with divalent metals, and the resulting structures have been used for isoniazid, doxorubicin, nifedipine, and ibuprofen delivery [138,139,140,141]. On the other hand, cationic chitosan has been reported to form ionic self-assemblies by Coulombic interaction with various anionic molecules. The ionic interaction produces self-assembled delivery systems with well-defined shapes and dimensions (nano/micro-particles, hydrogels and films, Table 1. The other factor can be hydrophobic interactions caused by various hydrophobically modified polysaccharides, where the hydrophobic groups range from acetyl groups to lipid molecules [149,153,154,159,164]. The hydrophobized carrier might effectively adsorb and deliver the target guest via supramolecular chemistry [181]. Cross-linkers such as glutaraldehyde [165], epichlorohydrin [179], and 1,3-diamino-propane [180] can produce irreversible supramolecular architectures, which are also prepared from co-polymer structures with polyacrylamide [168], polylactic acid [171], and poly-ε-caprolactone [177]. Throughout physical and chemical forces, various carbohydrate architectures are observed such as microspheres, nanoparticles, films and hydrogels, which properties and advantages for the representative drug delivery system are discussed in the following subsections.

#### 4.2.1. Nano-Particles (Spheres)

Nanoparticle entrapping drugs can penetrate cells and tissue gaps, and be delivered to the target organ with their nanosized dimensions. Particularly, polysaccharide-based nanoparticles control release profiles based on the biodegradability and stimuli (pH or temperature)-responsive structural changes [182,183]. The major problems such as the cytotoxicity and degradation products of nanoparticles can also be solved based on their biocompatibility and biodegradability. The hydrophilic groups (hydroxyl, amino, and carboxyl groups) in polysaccharides provide bio-adhesive properties forming non-covalent bonds with biological tissues. Furthermore, some polysaccharides have recognition ability on specific cell types, allowing targeted delivery. These materials have the additional potential to combine diagnosis and therapy, and can be applied to nanomedicine. In this respect, polysaccharide (mostly dextran or chitosan)-coated magnetic nanoparticles have been attractive for drug delivery owing to their improved drug absorption, their prolonged blood residence time and target-specific delivery [184].

#### 4.2.2. Microspheres

Polysaccharide-based microspheres can encapsulate many types of drugs, localize delivery of drug, and are easily administered through a syringe needle [185]. They have been commonly developed for treating many respiratory diseases, and the microsphere size is decisive for the release rate of drugs. The drug-polysaccharide supramolecular interactions and porosity may be other important factors. For pulmonary delivery, large particles may deposit in the respiratory tract before the target site, whereas small particles can aggregate before reaching the desired location. Depending on the polysaccharide species or molecular weight, the average particle sizes are varied by changing the solution viscosity [186]. The preparation methods for microsphere using polysaccharides are solvent extraction [187], spray-drying [188], emulsion-crosslinking [166,179,189] and precipitation [190]. The conventional preparation methods have also been modified for property improvement. In the spray drying method, good sphericity and a narrow size distributions could be obtained, however the burst effect and fast drug release need to be solved. Using the w/o/w emulsion-spray drying method, famotidine was released for several hours with a sustained type from chitosan microsphere, and wetting agents could be utilized to increase the release rate [191].

#### 4.2.3. Membrane (Film)

Membranes are mainly designed to control the release rate in transdermal delivery of drugs, and the applied materials can be used as tablet coatings, patches and wound dressings. The bio-adhesive and film-forming properties of polysaccharides are helpful for layer-by-layer type supramolecular structures [192]. Multilayers for the controlled delivery of drugs have advantages in terms of loading multiple components into interlayers and releasing the drugs sequentially layer-by-layer [193]. Although these films are regarded as more appropriate for hydrophilic drugs than hydrophobic ones [194,195], chitosan films were fabricated and used for local delivery of paclitaxel, and the biodegradation by lysozyme was used to control the drug release [196].

#### 4.2.4. Hydrogels

Hydrogels are water-swollen and hydrophilic membranes with pseudo-plastic properties that absorb a high amount of water to maintain their shape. Water can penetrate into the interstitial spaces of the three-dimensional polysaccharide network, providing hydrogel-like artificial tissues [197]. The highly porous structure allows loading drugs into the gel matrix and subsequent drug release [198]. Furthermore, the physical properties are elastically active and have low interfacial tension in biological fluids. The drug release from hydrogels can be controlled by swelling, diffusion, and degradation [199]. As well as, the charge distribution and chemical modification in polysaccharide structures contributes to it along with the surface state or porosity. In many cases, since polysaccharides are resistant to degradation by gastric and intestinal bacteria and susceptible to digestion by colonic microbial flora, they have been studied as colon-specific drug delivery systems [200].

## 5. Conclusions

Supramolecular complexation of carbohydrates and poorly soluble drugs efficiently enhances the bioavailability of drugs. In the structure of carbohydrates, –OH and –CH groups provide the sites for hydrogen bonding and hydrophobic interactions, respectively. Furthermore, various modified carbohydrates can expand the scope of available drugs as well as the architecture. With monosaccharides, the targeted delivery of drugs was mainly considered. Typical binary complexes using linear and cyclic oligosaccharides were developed for poorly soluble drugs as two-dimensional systems, and the aqueous drug solubility was clearly enhanced by the binary system. In the intermediate version, tertiary complexes with another factor could reinforce the effect. Further, multiple complexes and polysaccharide-based supramolecules were recently studied as three-dimensional drug delivery systems. So far, various combinatorial hybrid materials of carbohydrates have been successfully developed for advanced drug carrier systems. Therefore, novel materials based on carbohydrates will be promising for advanced applications in the biomedical and pharmaceutical fields. Further, these works will improve micro/nano fabrication technology, bio-mimetics, and tissue engineering based on the supramolecular carbohydrate architecture.
